# Identification of *cis*-Acting Elements and Splicing Factors Involved in the Regulation of *BIM* Pre-mRNA Splicing

**DOI:** 10.1371/journal.pone.0095210

**Published:** 2014-04-17

**Authors:** Wen Chun Juan, Xavier Roca, S. Tiong Ong

**Affiliations:** 1 Cancer and Stem Cell Biology Signature Research Programme, Duke-NUS Graduate Medical School, Singapore, Singapore; 2 School of Biological Sciences, Nanyang Technological University, Singapore, Singapore; 3 Department of Haematology, Singapore General Hospital, Singapore, Singapore; 4 Department of Medical Oncology, National Cancer Centre, Singapore, Singapore; 5 Division of Medical Oncology, Department of Medicine, Duke University Medical Center, Chapel Hill, North Carolina, United States of America; International Centre for Genetic Engineering and Biotechnology, Italy

## Abstract

Aberrant changes in the expression of the pro-apoptotic protein, BCL-2-like 11 (BIM), can result in either impaired or excessive apoptosis, which can contribute to tumorigenesis and degenerative disorders, respectively. Altering *BIM* pre-mRNA splicing is an attractive approach to modulate apoptosis because BIM activity is partly determined by the alternative splicing of exons 3 or 4, whereby exon 3-containing transcripts are not apoptotic. Here we identified several *cis*-acting elements and splicing factors involved in *BIM* alternative splicing, as a step to better understand the regulation of *BIM* expression. We analyzed a recently discovered 2,903-bp deletion polymorphism within *BIM* intron 2 that biased splicing towards exon 3, and which also impaired BIM-dependent apoptosis. We found that this region harbors multiple redundant *cis*-acting elements that repress exon 3 inclusion. Furthermore, we have isolated a 23-nt intronic splicing silencer at the 3′ end of the deletion that is important for excluding exon 3. We also show that PTBP1 and hnRNP C repress exon 3 inclusion, and that downregulation of PTBP1 inhibited BIM-mediated apoptosis. Collectively, these findings start building our understanding of the *cis*-acting elements and splicing factors that regulate *BIM* alternative splicing, and also suggest potential approaches to alter *BIM* splicing for therapeutic purposes.

## Introduction

The proper regulation of programmed cell death or apoptosis is critical to normal development, tissue homeostasis, and immune function [1]. Consequently, dysregulated apoptosis contributes to multiple disease states, including conditions associated with excessive cell death such as degenerative conditions, sepsis, and post-ischemic injuries, as well as impaired death such as autoimmune disease and cancer [2]. Both extracellular and intracellular signals contribute to the regulation of apoptosis, which in both instances results in the activation of certain proteases, known as caspases, that bring about cell destruction. Apoptosis mediated by the intracellular pathway (also known as the mitochondrial pathway) is orchestrated by members of the B-cell CLL/lymphoma 2 (BCL2) family of proteins. The BCL2 family includes both pro- and anti-apoptotic members which, via their relative levels of expression, determine whether a cell will live or die [3].

One prominent pro-apoptotic BCL2 family member, the BCL2-like 11 protein (also known as BIM, BCL2-Interacting Mediator of cell death), has been intensively studied because of its pivotal role in promoting mitochondrial-mediated apoptosis under both physiological and pathological conditions. Physiologically, BIM induces apoptosis by opposing the pro-survival members of the family such as BCL2, or by directly binding to and activating pro-apoptotic effectors such as BCL2-associated X protein (BAX) [4]. Pathologically, alterations in BIM expression are associated with several diseases. For example, BIM is commonly downregulated in cancer, while its upregulation is necessary for sensitivity to cancer therapy-induced apoptosis [5]–[10]. Furthermore, increased BIM expression has been shown to contribute to increased cardiomyocyte and neuronal cell death following ischemia [11], [12], as well as neuronal cell death in Alzheimer's disease [13], while decreased BIM expression confers protection from viral-induced hepatitis and sepsis-related mortality [14], [15]. Accordingly, there has been intense interest to understand how BIM expression and function are physiologically regulated, and how these processes might be therapeutically modulated to enhance or attenuate BIM-mediated cell death.

In addition to transcriptional and post-translational regulation of BIM, recent work has highlighted important contributions from epigenetic regulation [6] as well as alternative splicing [6], [16]–[18]. Alternative splicing is the process by which precursor mRNAs (pre-mRNAs) are spliced differentially, leading to distinct mRNA and protein isoforms, thus increasing the diversity of the human transcriptome and proteome [19]. Often the protein isoforms generated by alternative splicing have considerable functional differences, and many such isoforms can contribute to human disease, including cancer [20]. Alternative splicing is regulated by *cis*-acting elements within pre-mRNAs and *trans*-acting factors. The essential *cis*-acting elements are the 5′ splice site, the 3′ splice site, as well as the branchpoint sequence, which conform to partially conserved motifs that are recognized by cognate *trans*-acting factors [21]. Additional *cis*-acting elements that regulate alternative splicing include exonic or intronic splicing enhancers and silencers (ESEs, ISEs, ESSs, ISSs), which respectively activate or repress use of particular splice sites or exon inclusion [22], [23]. *Trans*-acting factors regulate alternative splicing by associating with *cis*-acting elements, and include serine/arginine (SR)-rich proteins as well as heterogeneous nuclear ribonucleoproteins (hnRNPs) [23], [24].

As mentioned, *BIM* alternative splicing provides another level of control over BIM function. Mechanistically, the alternative inclusion of either *BIM* exons 3 or 4 gives rise to two distinct groups of mRNA isoforms. Exons 3 and 4 cannot be spliced together because exon 3 contains a functional polyadenylation (pA) signal and lacks a bona-fide 5′ splice site. Exon 3-containing splice variants are not pro-apoptotic because they lack the BH3 domain encoded in exon 4, which is required for interacting and antagonizing the pro-survival members of the BCL2 family [16], [17], [25]. Both *cis*-acting elements and *trans*-acting factors contribute to *BIM* splicing. For example, recent work suggests that single nucleotide polymorphisms (SNPs) within *BIM* influence its splicing in *cis*. Specifically, a C>T SNP (rs724710) in *BIM* exon 4 has been shown to affect the inclusion of *BIM* exon 3, and may contribute to drug resistance in acute lymphoblastic leukemia [18]. Aberrant *BIM* splicing has also been observed in breast tumors driven by the splicing factor SRSF1. Here, SRSF1 overexpression promotes the inclusion of exon 3 over exon 4, which in turn favors the expression of non-apoptotic splice variants of *BIM* [17]. Accordingly, *BIM* alternative splicing is emerging as an important mediator of dysregulated cell death in human disease.

In our studies aimed at identifying genetic causes for resistance to tyrosine kinase inhibitors (TKIs) in patients diagnosed with chronic myelogenous leukemia (CML), we discovered a 2,903-bp deletion polymorphism within *BIM* intron 2 that was strongly associated with this resistance. We experimentally demonstrated that the deletion biased *BIM* splicing toward exon 3 instead of exon 4, which impaired BIM-dependent cell death upon TKI treatment [16]. These observations suggested the presence of functionally important *cis*-acting elements within the 2,903-nt fragment that repress *BIM* exon 3 splicing. In this current study, we set out to identify *cis*-acting elements and *trans*-acting factors involved in *BIM* alternative splicing, to better understand *BIM* regulation in the context of TKI-resistance and other physiopathological conditions. Intriguingly, we found that the polymorphic fragment contains multiple *cis*-acting elements that limit exon 3 inclusion. Despite a high level of redundancy, we were able to identify a 23-nt ISS at the 3′ end of the polymorphic fragment that is important for excluding exon 3. Further, we found that a GGGG motif and two poly-U tracts within the 23-nt ISS plays a critical role in silencing exon 3. We also identified *trans*-acting factors that repress inclusion of endogenous *BIM* exon 3, thereby protecting cells from therapy-induced apoptosis. Taken together, our findings provide important insights into how functionally relevant *BIM* isoforms are generated by alternative splicing, and also suggest approaches by which BIM-mediated cell death might be modulated for therapeutic gain. Such approaches include the use of antisense oligonucleotides (ASOs) that bind to and inhibit the activity of *cis*-acting elements, as well as drugs that modulate the expression of splicing factors to alter *BIM* splicing.

## Materials and Methods

### Cell culture

The CML cell lines, K562 and KCL22, were obtained from the German Collection of Microorganisms and Cell Cultures. These cell lines were cultured in Roswell Park Memorial Institute (RPMI) 1640 medium (Gibco) supplemented with penicillin/streptomycin (Hyclone*)*, glutamine (Hyclone), 10% fetal bovine serum (Hyclone), and were incubated in a humidified incubator at 37°C with 5% CO_2_.

### Minigene plasmids construction

**Figure 1:**
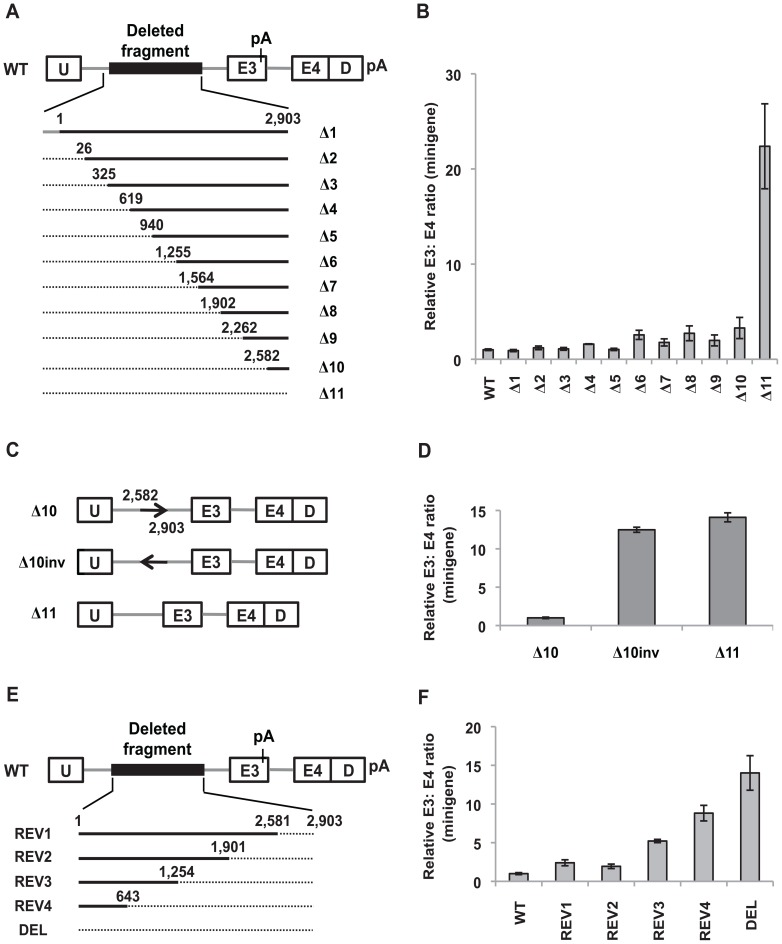
The 2,903-nt polymorphic fragment contains redundant *cis*-acting elements that repress inclusion of *BIM* exon 3. (A) Schematic of the forward deletion mutants within the context of the WT minigene. Exons are depicted as open boxes, introns as grey lines, and the polymorphic fragment is indicated as a black box. Exons and introns are not drawn to scale. The minigene harbors two adenovirus exonic sequences, U and D. The pA signal is present in *BIM* exon 3 and downstream of D. Whereas deleted sequences are denoted by dotted lines, retained sequences are denoted by solid black lines. The numbers above each solid black line indicate the boundaries of the retained sequences. (B) Real-time RT-PCR analysis of RNA from K562 cells nucleofected with the indicated forward deletion constructs to determine the ratio of U-E3 to U-E4-D transcripts. Three biological replicates were performed and the relative minigene E3: E4 ratio was determined by normalizing to the E3: E4 ratio of K562 cells nucleofected with the WT minigene. Error bars represent ± standard error of the mean (SEM). (C) Schematic of the Δ10inv minigene that harbors an inversion of +2,582 to +2,903 of the polymorphic fragment. (D) Real-time RT-PCR analysis of RNA from K562 cells nucleofected with the minigenes described in (C) to assess the ratio of exon 3- to exon 4-containing minigene products. The relative minigene E3: E4 ratio was determined by normalizing to the E3: E4 ratio of K562 cells nucleofected with Δ10. (E) Schematic of the reverse deletion mutants within the context of the WT minigene. (F) Real-time RT-PCR analysis of RNA from K562 cells nucleofected with the indicated reverse deletion constructs to assess the ratio of exon 3- to exon 4-containing minigene products. Three biological replicates were performed and the relative minigene E3: E4 ratio was determined by normalizing to the E3: E4 ratio of K562 cells nucleofected with the WT minigene.

**Figure 2:**
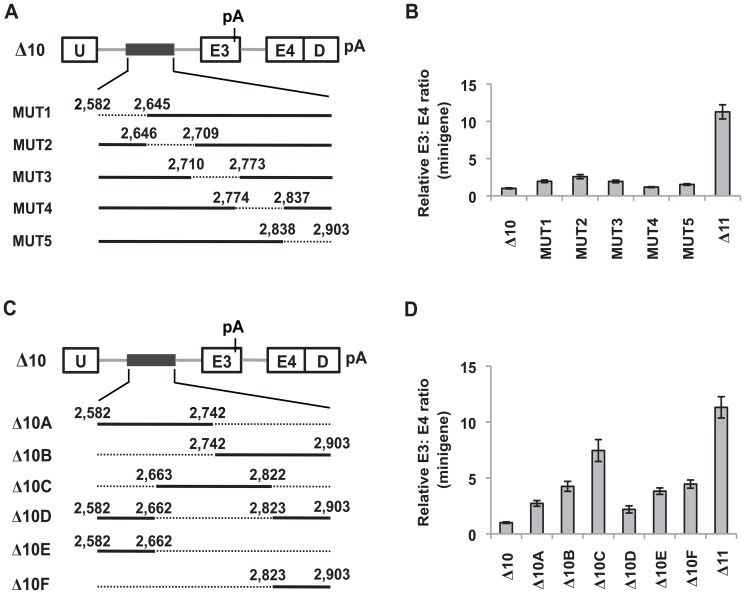
*Cis*-acting elements regulating splicing of exon 3 within +2,582 to +2,903 of the polymorphic fragment. (A) Schematic diagram of the 64-nt internal deletions in the context of the Δ10 minigene. Whereas deleted sequences are denoted by dotted lines, retained sequences are denoted by solid black lines. The numbers above each dotted line indicate the boundaries of the removed sequences. (B) Real-time RT-PCR analysis of RNA from K562 cells nucleofected with the indicated deletion constructs to determine the ratio of exon 3- to exon 4-containing minigene products. The Δ11 minigene serves as a positive control for enhanced exon 3 inclusion as it does not contain any sequences from the polymorphic fragment that repress exon 3. Results are presented as an average of three biological replicates and the relative minigene E3: E4 ratio was determined by normalizing to the E3: E4 ratio of K562 cells nucleofected with the Δ10 minigene. Error bars represent ± SEM. (C) Schematic diagram of the deletions in the context of the Δ10 minigene. Whereas deleted sequences are denoted by dotted lines, retained sequences are denoted by solid black lines. The numbers above each solid black line indicate the boundaries of the retained sequences. (D) Real-time RT-PCR analysis of RNA from K562 cells nucleofected with the constructs described in (C) to assess the ratio of minigene products containing either exon 3 or exon 4. The Δ11 minigene serves as a positive control for enhanced exon 3 inclusion. Results are presented as an average of triplicates and the relative minigene E3: E4 ratio was determined by normalizing to the E3: E4 ratio of K562 cells nucleofected with Δ10. Error bars represent ± SEM.

**Figure 3:**
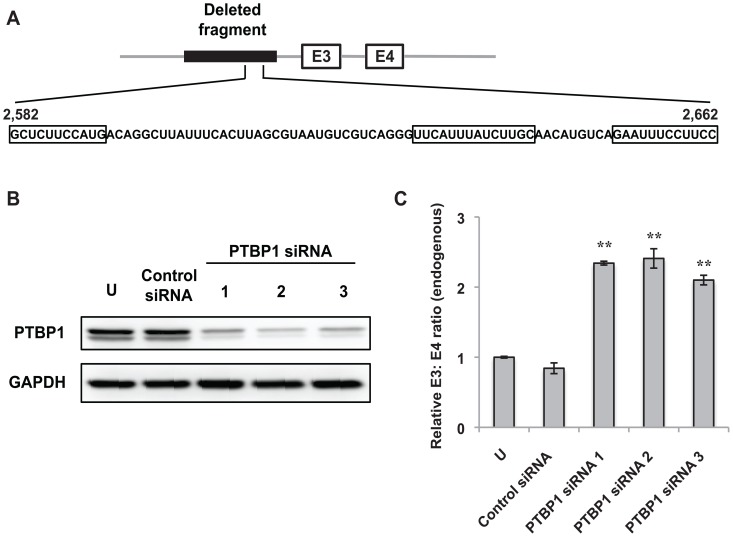
PTBP1 represses inclusion of endogenous *BIM* exon 3. (A) +2,582 to +2,662 of the polymorphic fragment has been expanded to show the nucleotide sequence. The three predicted PTBP1 binding sites are boxed. (B) Western blot analysis for PTBP1 protein levels after nucleofecting K562 cells with three different PTBP1-specific siRNA duplexes. Equal loading in each lane was determined by blotting of glyceraldehyde 3-phosphate dehydrogenase (GAPDH). “U” represents control cells that were not subjected to nucleofection. (C) Real-time RT-PCR analysis of RNA from K562 cells nucleofected with either control or PTBP1-specific siRNA duplexes to determine the ratio of endogenous exon 3- to exon 4-containing *BIM* transcripts. “U” represents control cells that were not subjected to nucleofection. Results are presented as an average of three biological replicates and the relative endogenous *BIM* E3: E4 ratio was determined by normalizing to the E3: E4 ratio of K562 cells that were not nucleofected. Error bars represent ± SEM. **p<0.01.

**Figure 4:**
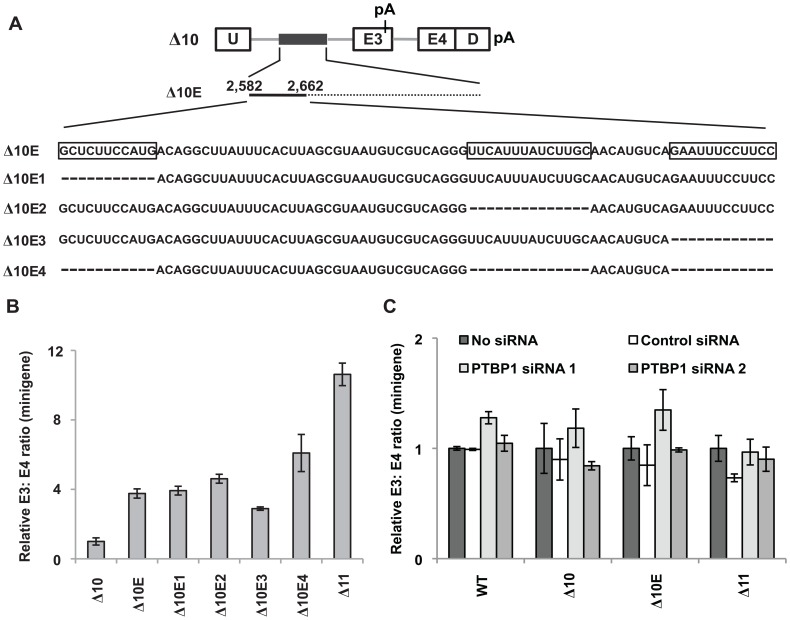
PTBP1 represses inclusion of *BIM* exon 3 independently of the 2,903-nt polymorphic fragment. (A) Schematic of the deletions made on the Δ10E minigene to remove the putative PTBP1 binding sites. +2,582 to +2,662 of the polymorphic fragment has been expanded to show the nucleotide sequence. The predicted PTBP1 binding sites on Δ10E are boxed, whereas the deletions are indicated by dashes. (B) Real-time RT-PCR analysis of RNA from K562 cells nucleofected with the minigene constructs described in (A) to assess the ratio of exon 3- to exon 4-containing minigene products. The Δ11 minigene serves as a positive control for enhanced exon 3 inclusion. Results are presented as an average of triplicates and the relative minigene E3: E4 ratio was determined by normalizing to the E3: E4 ratio of K562 cells nucleofected with the Δ10 minigene. Error bars represent ± SEM. (C) K562 cells were either nucleofected with control or PTBP1-specific siRNA duplexes. 24 hours later, these cells were nucleofected with either the WT, Δ10, Δ10E or Δ11 minigene. Real-time RT-PCR analysis of RNA from these cells was performed after another 24 hours to determine the ratio of exon 3- to exon 4-containing minigene products. Results are presented as an average of triplicates and the relative minigene E3: E4 ratio was determined by normalizing to the E3: E4 ratio of K562 cells nucleofected with the same minigene, but without any siRNA. Error bars represent ± SEM.

**Figure 5:**
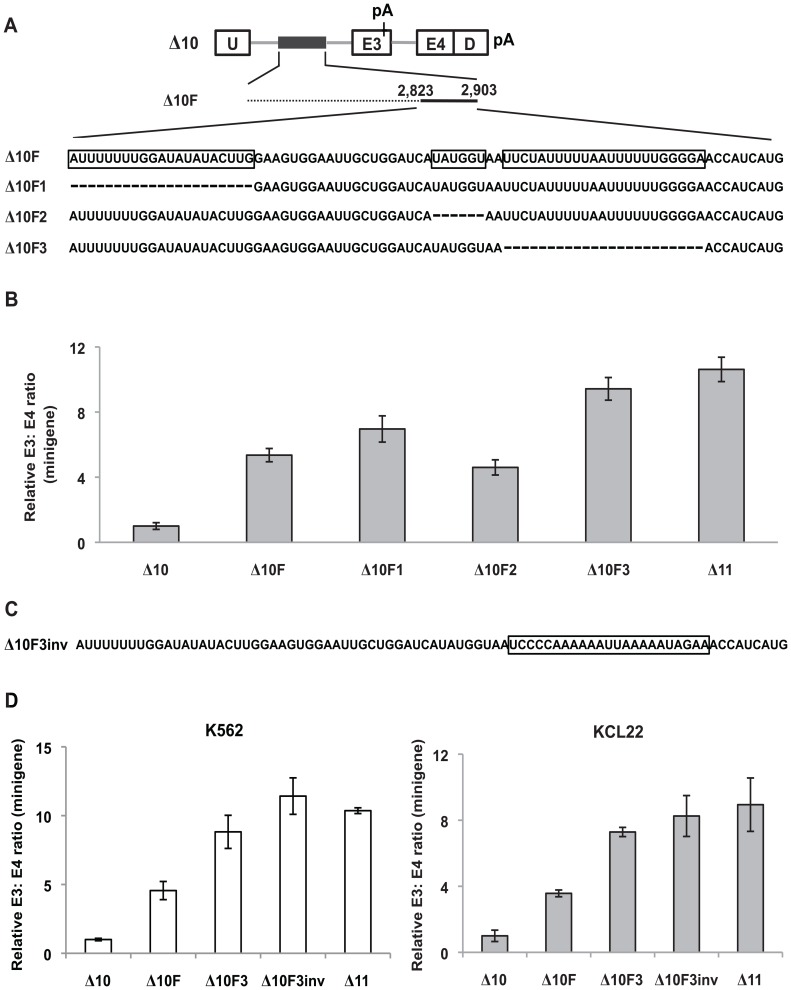
Identification of a 23-nt ISS within +2,823 to +2,903 of the polymorphic fragment. (A) Schematic diagram of the deletions made on the Δ10F minigene to remove the predicted ISSs. +2,823 to +2,903 of the polymorphic fragment has been expanded to reveal the nucleotide sequence. The predicted ISSs on Δ10F are boxed, whereas deletions made to the sequence are indicated by dashes. (B) Real-time RT-PCR analysis of RNA from K562 cells nucleofected with the constructs in (A) to determine the ratio of exon 3- to exon 4-containing minigene products. The Δ11 minigene serves as a positive control for enhanced exon 3 inclusion. Results are presented as an average of triplicates and the relative minigene E3: E4 ratio was determined by normalizing to the E3: E4 ratio of K562 cells nucleofected with Δ10. Error bars represent ± SEM. (C) The Δ10F3inv minigene is generated in the context of the Δ10F minigene, in which the putative 23-nt ISS has been inverted. The inversion mutation is boxed. (D) Real-time RT-PCR analysis of RNA from K562 and KCL22 cells that were nucleofected with the indicated minigene constructs to assess the ratio of exon 3- to exon 4-containing minigene products. Results are presented as an average of three biological replicates and the relative minigene E3: E4 ratio was determined by normalizing to the E3: E4 ratio of K562 and KCL22 cells nucleofected with Δ10. Error bars represent ± SEM.

**Figure 6:**
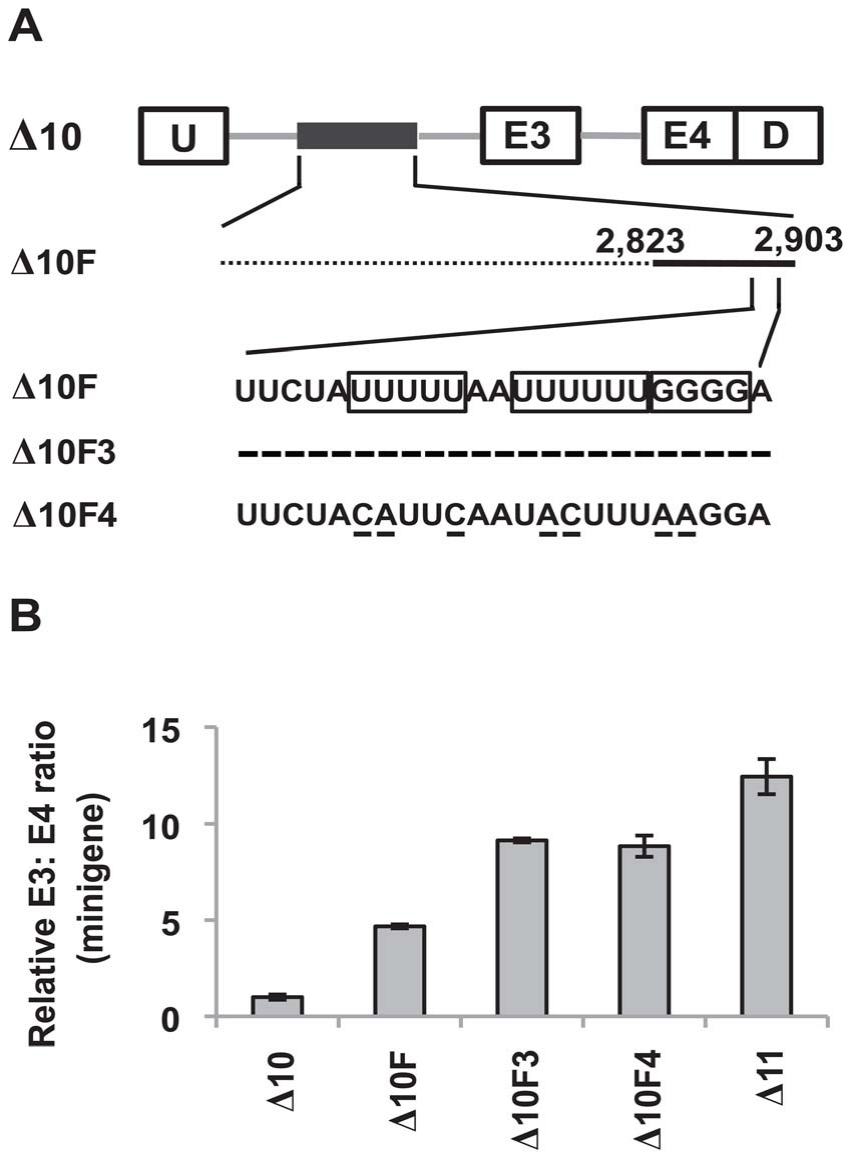
Identification of motifs within the 23-nt ISS that are essential for repressing exon 3 inclusion. (A) Schematic diagram of the Δ10, Δ10F, Δ10F3 and Δ10F4 minigenes. The 23-nt ISS has been expanded to show the nucleotide sequence. The GGGG motif and the poly-U tracts in Δ10F are boxed. Removal of the 23-nt ISS in Δ10F3 is indicated by dashes. Point mutations in Δ10F4 that disrupt the GGGG motif and the poly-U tracts are underlined. (B) Real-time RT-PCR analysis of RNA from K562 cells nucleofected with the minigene constructs shown in (A) to determine the ratio of exon 3- to exon 4-containing minigene products. The Δ11 minigene serves as a positive control for enhanced exon 3 inclusion. Results are presented as an average of three biological replicates and the relative minigene E3: E4 ratio was determined by normalizing to the E3: E4 ratio of K562 cells nucleofected with the Δ10 minigene. Error bars represent ± SEM.

**Figure 7:**
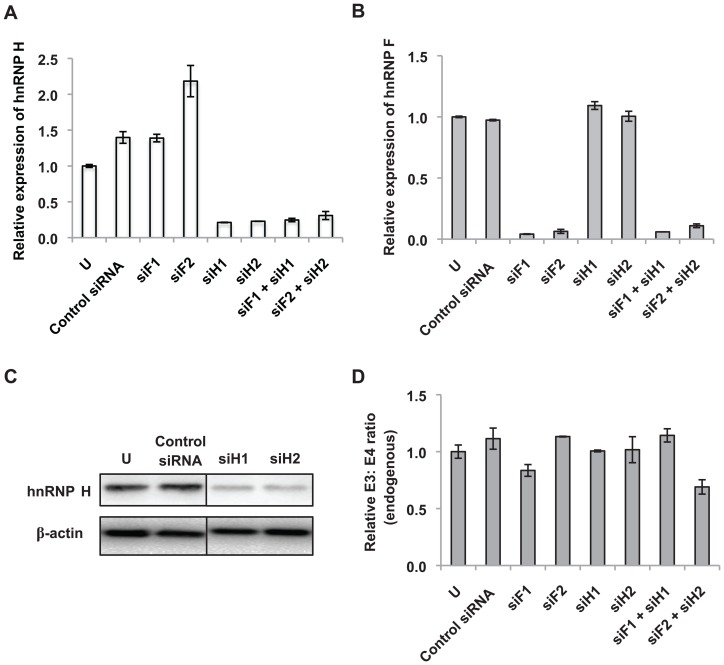
Knockdown of hnRNP H and hnRNP F does not promote inclusion of *BIM* exon 3. K562 cells were nucleofected with two different siRNA duplexes targeting either hnRNP H (siH1, siH2) or hnRNP F (siF1, siF2). Some cells were also nucleofected with siRNA duplexes targeting both hnRNP H and F (siF1 and siH1, siF2 and siH2). 48 hours later, real-time RT-PCR analysis of RNA from K562 cells was performed to determine the relative expression of (A) hnRNP H and (B) hnRNP F transcripts. “U” represents control cells that were not subjected to nucleofection. Results are presented as an average of three biological replicates and data is shown as mean ± SEM relative to non-nucleofected cells. (C) Western blot showing the efficacy of the two siRNA duplexes targeting hnRNP H. Protein bands shown are from different lanes but within the same membrane. Equal loading in each lane was determined by blotting of β-actin. (D) Ratio of endogenous *BIM* transcripts harboring exon 3 over exon 4 after nucleofecting K562 cells with the indicated siRNAs. “U” represents control cells that were not subjected to nucleofection. Results are presented as an average of three biological replicates and the relative endogenous E3: E4 ratio was determined by normalizing to the endogenous E3: E4 ratio of K562 cells that were not nucleofected with siRNA. Error bars represent ± SEM.

**Figure 8:**
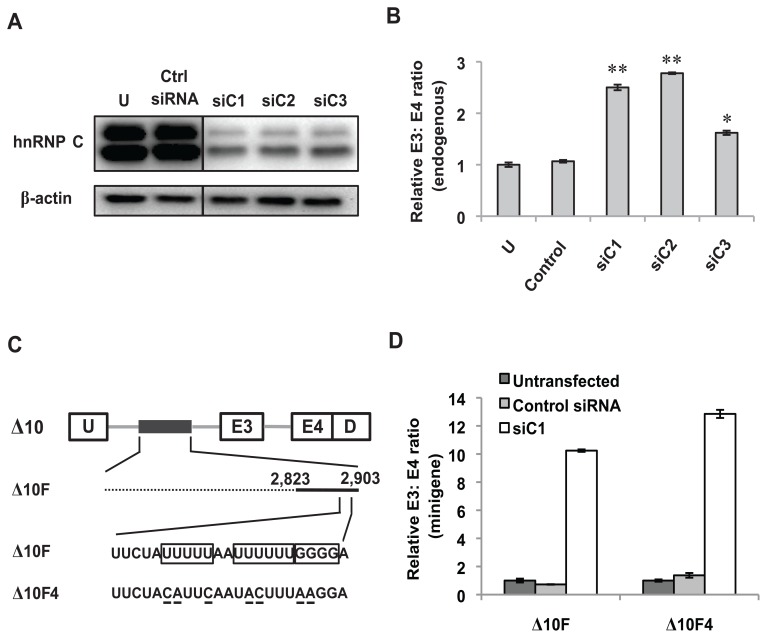
HnRNP C regulates exon 3 inclusion independently of the GGGG motif and the poly-U tracts. (A) Western blot demonstrating the knockdown of hnRNP C using three different siRNA duplexes (siC1, siC2, siC3). Protein bands shown are from different lanes but within the same membrane. (B) Real-time RT-PCR analysis of RNA from K562 cells nucleofected with either control or hnRNP C-specific siRNA duplexes to assess the ratio of endogenous exon 3- to exon 4-containing *BIM* transcripts. “U” represents control cells that were not subjected to nucleofection. Results are presented as an average of three biological replicates and the relative endogenous E3: E4 ratio was determined by normalizing to the endogenous E3: E4 ratio of K562 cells that were not nucleofected with siRNA. Error bars represent ± SEM. *p<0.05, **p<0.01. (C) Schematic diagram of the Δ10, Δ10F and Δ10F4 minigene constructs. The 23-nt ISS has been expanded to show the nucleotide sequence. The GGGG motif and the poly-U tracts in Δ10F are boxed. Point mutations in Δ10F4 that disrupt the GGGG motif and the poly-U tracts are underlined. (D) K562 cells were nucleofected with either control or hnRNP C-specific siRNA (siC1). 24 hours later, these cells were nucleofected with either the Δ10F or the Δ10F4 minigene. After another 24 hours, real-time RT-PCR was performed on RNA from these cells to determine the ratio of exon 3- to exon 4-containing minigene products. Results are presented as an average of three biological replicates and the relative minigene E3: E4 ratio was determined by normalizing to the E3: E4 ratio of K562 cells nucleofected with the same minigene, but without any siRNA. Error bars represent ± SEM.

Overlap extension PCR was performed to generate PCR products with 5′ and 3′ restriction sites for EcoRV and ClaI, respectively (primer sequences available on request). The previously described WT minigene [16] served as a template to generate PCR products harboring deletions and inversions within the 2,903-nt polymorphic fragment described in Figure 1. The Δ10, Δ10E or Δ10F minigene was used as the template to generate PCR products harboring deletions, inversions or mutations within +2,582 to +2,903 of the polymorphic fragment described in Figures 2–8. PCR products were purified using QIAquick gel extraction kit (Qiagen) and cloned into EcoRV- and ClaI-digested WT minigene.

### Real-time RT-PCR

Total RNA was extracted using the RNeasy Mini Kit (Qiagen). RNA was reverse transcribed using Superscript III First-Strand Synthesis System (Invitrogen) at a volume of 20 µl. Quantification of endogenous and minigene transcripts was assessed using the iQ5 Multicolor Real-Time Detection System (Bio-rad) with a reaction volume of 25 µl. Primers were annealed at 59°C for 20 seconds and the amplicon was extended at 72°C for 30 seconds, for 40 cycles. Transcript levels of β-actin or the adenovirus exonic sequence (U) were used to normalize endogenous and minigene transcripts, respectively. The following primers were used: hnRNP F (forward: 5′-AATTGTGCCAAACGGGATCAC-3′; reverse: 5′-GTGTTTCCCTAGAGCCTTCTC-A-3′), hnRNP H (forward: 5′-ATTCAAAATGGGGCTCAAGGTAT-3′; reverse: 5′-GTGTCAGG-

ACTATTTGGACCAG-3′). Primer sequences to amplify endogenous exon 3- and exon 4- containing *BIM* transcripts, β-actin and minigene transcripts (U-E3 and U-E4) have been described previously [16].

### Nucleofection

1×10^6^ K562 or KCL22 cells were transiently nucleofected with 1 µg of each minigene using the Cell Line Nucleofector Kit V (Lonza). RNA was extracted from these cells after 24 hours. For knockdown studies, siRNAs were synthesized either from Integrated DNA Technologies or from Ambion. The siRNA sequences were: control siRNA (5′-GGUCCGGCUCCC-CCAAAUC-3′) [26], PTBP1 siRNA 1 (5′-CUUCCAUCAUUCCAGAGAA-3′) [26],

PTBP1 siRNA 2 (5′- CAAAGCCUCUUUAUUCUUU-3′), PTBP1 siRNA 3 (5′-CAGUUUACCU-GUUUUUAAA-3′) hnRNP H (siH1: 5′-GAAGCAUACUGGUCCAAAU-3′; siH2: 5′-GGAUUU-GGGUCAGAUAGAU-3′), hnRNP F (siF1: 5′-GCAGCACAGAUAUAUAGAA-3′; siF2: 5′-CGA-CCGAGAACGACAUUUA-3′) and hnRNP C (siC1: 5′-CAACGGGACUAUUAUGAUA-3′;

siC2: 5′-GAUGAAGAAUGAUAAGUCA-3′; siC3: 5′-ACACUCUUGUGGUCAAGAA). Knockdowns were performed using siRNAs at 100 nM concentration on 1×10^6^ K562 cells using the Cell Line Nucleofector Kit V (Lonza).

### Western blot

Whole cell lysates were obtained using RIPA lysis buffer (Millipore). Lysis was performed on ice for 20 minutes in the presence of protease and phosphatase inhibitors (Roche). Protein concentration was estimated using the Bradford assay (Bio-Rad Laboratories). The samples were resolved by electrophoresis in a 11% polyacrylamide gel. Once electrophoresis was completed, the gel was rinsed in water before the proteins were electro-blotted onto a polyvinylidene difluoride membrane. Subsequently, the blot was blocked for one hour in 5% (w/v) non-fat milk in Tris buffered saline-Tween (TBST) (100 mM Tris-base, 154 mM NaCl, 0.1% (v/v) Tween 20, pH 7.4). The following primary antibodies were used to probe the membrane: β-actin (#AC-15, Sigma), PTBP1 (32-5000, Invitrogen), hnRNP H (ab10374, Abcam), hnRNP C (ab10294, Abcam), BIM (Cell Signaling Technology, #2819), caspase 3 (Cell Signaling Technology, #9662), PARP (Cell Signaling Technology, #9542) and GAPDH (Cell Signaling Technology #2118). All primary antibodies were diluted at 1∶1000 except for β-actin (1∶5000) in TBST with 5% (w/v) non-fat milk. Three washes in TBST were performed after primary antibody incubation before the appropriate horse radish peroxidase-conjugated secondary antibodies (Santa Cruz Biotechnology) at a dilution of 1∶5000 were used to probe the membrane. The membrane was visualized using the Western Lightning chemiluminescence reagent (PerkinElmer).

### Computational analysis to predict *cis*-acting elements that regulate *BIM* splicing

Prediction of *cis*-regulatory elements for splicing of *BIM* exon 3 was performed by using Sfmap [27], PESX [28], Human Splicing Finder [29] and Spliceaid [30]. Regulatory elements that were predicted by more than one program were selected for further functional analysis.

## Results

### The 2,903-nt polymorphic fragment within intron 2 of *BIM* contains multiple redundant *cis*-acting elements that repress *BIM* exon 3 splicing

To assess whether the 2,903-nt fragment contains regulatory elements that repress splicing of *BIM* exon 3, we previously created two *BIM* minigenes with (WT) and without (DEL) the polymorphic fragment fused to adenovirus exonic sequences U and D (Fig. 1A and 1E). The DEL minigene strongly favored the inclusion of exon 3 over exon 4 when compared to WT, thereby recapitulating the splicing of endogenous *BIM* transcripts [16]. Therefore, we were able to use the WT minigene to identify intronic splicing silencers (ISSs) within the 2,903-nt fragment that repress exon 3 inclusion.

To define these regulatory elements, we generated a series of forward sequential deletions in the 2,903-nt fragment in the context of the WT minigene (Fig. 1A, Δ1–Δ11). K562 cells were nucleofected with these constructs, and the ratio of U-E3 to U-E4-D transcripts was determined using real-time RT-PCR. Remarkably, all forward deletions removing most of the 2,903-nt failed to cause a substantial increase in exon 3 inclusion (Fig. 1B). The inclusion of exon 3 over exon 4 remained low even in the Δ10 minigene, which consist of only the last 322-nt from +2,582 to +2,903 of the polymorphic fragment. A very pronounced increase in exon 3 inclusion was observed in Δ11, as the last 322-nt were removed (Fig. 1B). To exclude the possibility that excessive shortening of the intron in Δ11 caused the significant increase in exon 3 inclusion, we created a size-matched control of Δ10 by substituting +2,582 to +2,903 of the deletion with an inversion of this sequence (Fig. 1C). This inversion (Δ10inv) led to an increase in exon 3 inclusion to almost the same extent as that of Δ11 (Fig. 1D). Thus, the large increase in exon 3 inclusion in Δ11 is not a result of an excessive truncation of the intron. These results also suggest that the +2,582 to +2,903 segment contains specific ISS(s) that repress exon 3 splicing.

We also analyzed a panel of reverse deletion constructs (Fig. 1E, REV1-REV4). Intriguingly, removing the last 322-nt in REV1 only resulted in a modest increase in exon 3 inclusion (Fig. 1F). Removing an additional 680-nt in REV2 failed to cause a significant change in exon 3 inclusion when compared to REV1. However, a greater increase in exon 3 inclusion was observed with additional reverse deletions (REV3, REV4 and DEL, Fig. 1F). Taken together, these results suggest that the 2,903-nt polymorphic fragment contains multiple redundant *cis*-acting elements that repress inclusion of exon 3, and that these elements are distributed throughout the fragment. In addition, the data also indicate that the last 322-nt, comprising +2,582 to +2,903 of the polymorphic fragment, contain ISS(s) that are sufficient but not necessary for repressing exon 3. Despite this extensive redundancy, we sought to further elucidate the *cis*-regulatory elements within the polymorphic fragment. To this end, we focused on the elements within the +2,582 to +2,903 segment at the 3′ end of the deletion fragment.

### +2,582 to +2,662 and +2,823 to +2,903 of the polymorphic fragment contain most of the splicing silencers at the 3′ end of the deletion that repress *BIM* exon 3

To characterize the ISS(s) within +2,582 to +2,903 of the polymorphic fragment, we introduced internal deletions within this region of approximately 64-nt in the context of the Δ10 minigene (Fig. 2A). Interestingly, most of these internal deletions resulted in a very modest increase in exon 3 inclusion when compared with Δ11 (which lacks any sequence from the polymorphic fragment) (Fig. 2B). In addition, removing +2,774 to +2,837 of the polymorphic fragment (MUT4) did not lead to an increase in exon 3 inclusion. These results suggest that there are also multiple elements within +2,582 to +2,903 that limit inclusion of exon 3.

Because the internal deletions described above failed to result in a striking increase in exon 3 inclusion, we generated another panel of minigenes harboring larger deletions of at least 160-nt in the context of Δ10 (Fig. 2C). Consistent with the earlier results, removing half of the sequence on either end of the remaining 322-nt (Δ10A and Δ10B) resulted in an increase in exon 3 inclusion, further indicating that multiple elements that repress splicing of exon 3 are found along the +2,582 to +2,903 segment (Fig. 2D). Remarkably, removing the first and last 81-nt from the remaining 322-nt (Δ10C), while retaining +2,663 to +2,822 of the deletion, resulted in a greater increase in exon 3 inclusion which was the closest to the inclusion levels in Δ11 (Fig. 2D). The increase in exon 3 inclusion was lower when +2,582 to +2,662 and +2,823 to +2,903 of the polymorphic fragment were retained in the minigene (Δ10D) when compared to the Δ10A and the Δ10B minigenes (Fig. 2D). Collectively, these observations indicate that +2,582 to +2,662 and +2,823 to +2,903 of the polymorphic fragment – the first and last 81 nts – contain most, but not all, of the ISS(s) at the 3′ end of the polymorphic fragment that repress exon 3. Consistent with this observation, inclusion of exon 3 remained low when either +2,582 to +2,662 or +2,823 to +2,903 of the polymorphic fragment (Δ10E and Δ10F) was retained in the minigene (Fig. 2D).

### Polypyrimidine tract binding protein 1 (PTBP1) silences *BIM* exon 3 but not via the 2,903-nt polymorphic fragment

To further define elements within +2,582 to +2,662 of the polymorphic fragment that repress inclusion of *BIM* exon 3, we adopted a computational approach to predict splicing silencers. The +2,582 to +2,662 fragment contained three putative binding sites for PTBP1 (Fig. 3A), which is a well-known splicing repressor [31]–[33]. Therefore, we hypothesized that PTBP1 may repress the inclusion of *BIM* exon 3, and that it might do so via these three sites. To test these possibilities, we downregulated PTBP1 using three different siRNA duplexes. Western blotting showed that PTBP1 protein levels were greatly reduced upon siRNA knockdown (Fig. 3B). When PTBP1 expression was silenced, analysis of the endogenous *BIM* transcripts revealed that there was a 2-fold increase in the ratio of exon 3- to exon 4-containing transcripts (Fig. 3C). These results indicate that PTBP1 is a repressor of exon 3 inclusion.

To address whether the three putative PTBP1 binding sites within the +2,582 to +2,662 fragment are important for repressing exon 3 inclusion, we deleted them in the context of Δ10E, and predicted an increase in exon 3 splicing (Fig. 4A). Removing only one of the predicted PTBP1 binding sites (Δ10E1 and Δ10E2) did not result in a significant increase in exon 3 inclusion when compared to Δ10E (Fig. 4B). Unexpectedly, removing the third predicted PTBP1 binding site in the Δ10E3 minigene led to a modest decrease in exon 3 inclusion when compared to Δ10E (Fig. 4B). Because the putative PTBP1 binding sites may act redundantly, we generated the Δ10E4 minigene with all three binding sites removed (Fig. 4A). However, only a modest increase in exon 3 inclusion with respect to Δ10E was observed when K562 cells were nucleofected with Δ10E4 (Fig. 4B). Collectively, these results strongly suggest that the three predicted PTBP1 binding sites within +2,582 to +2,662 of the polymorphic fragment do not play a role in repressing exon 3 inclusion.

To further address whether PTBP1 regulates the inclusion of *BIM* exon 3 via the 2,903-nt polymorphic fragment, we silenced PTBP1 in K562 cells using siRNAs and nucleofected these cells with the WT, Δ10, Δ10E or Δ11 minigenes. In all constructs, silencing of PTBP1 failed to change exon 3 inclusion (Fig. 4C). These results indicate that PTBP1 does not regulate inclusion of exon 3 via +2,582 to +2,662 of the polymorphic fragment, which is consistent with the previous observation that removal of the three putative PTBP1 binding sites did not significantly change exon 3 inclusion. In addition, knockdown of PTBP1 did not significantly increase exon 3 inclusion in the WT minigene with an intact 2,903-nt fragment (Fig. 4C). From these results, we concluded that PTBP1 does not regulate inclusion of *BIM* exon 3 via the 2,903-nt polymorphic fragment. Because our minigenes do not contain all the sequences in introns 2 and 3, our results suggest that PTBP1 represses exon 3 via other *BIM* sequences, or that this regulation is indirect (see Discussion).

### +2,823 to +2,903 of the polymorphic fragment contains a 23-nt silencer that represses exon 3 inclusion

We next sought to identify the ISS(s) within +2,823 to +2,903 of the polymorphic fragment. *In silico* predictions suggested that there were three putative silencer regions within this fragment (Fig. 5A). To investigate whether these predicted silencer regions play a role in excluding exon 3, we removed them in the context of the Δ10F minigene (Δ10F1-3, Fig. 5A). Remarkably, removing a 23-nt sequence closest to the 3′ end of the deletion (Δ10F3) resulted in a dramatic increase in exon 3 inclusion when compared with Δ10F, an increase that was almost equivalent to that produced by Δ11 (Fig. 5B). In contrast, comparatively small splicing changes were detected when the other two predicted silencer regions (Δ10F1 and Δ10F2) were removed (Fig. 5B). To exclude the possibility that the increase in exon 3 inclusion in Δ10F3 was due to excessive shortening of the intron, we created a size-matched control (Δ10F3inv) with an inversion of the 23-nt putative silencer region (Fig. 5C). Strikingly, an inversion of the putative 23-nt silencer (Δ10F3inv) led to a similar or slightly higher increase in exon 3 inclusion than that of Δ10F3 in both K562 and KCL22 cells (Fig. 5D). The consistent results between these two cell lines exclude the possibility of cell line-specific effects. From these results, we conclude that +2,823 to +2,903 of the polymorphic fragment contains a 23-nt ISS, or more likely multiple ISSs, that is/are critical for repressing exon 3 inclusion. Furthermore, the 23-nt ISS does not appear to be specific to a particular cell line.

### Mutating the GGGG motif and the poly-U tracts within the 23-nt ISS enhance exon 3 inclusion

To further characterize the 23-nt ISS, we searched for the presence of binding motifs of splicing repressor proteins. The 23-nt ISS contains two poly-U tracts and a GGGG motif (Fig. 6A). Previous studies have shown that hnRNP C (among other proteins) associates with poly-U tracts [34], whereas hnRNP H and hnRNP F are two closely related proteins that bind G tracts [35]. Therefore, we hypothesized that the 23-nt ISS may repress the inclusion of *BIM* exon 3 through the involvement of the poly-U tracts and the GGGG motif. To test this, we introduced mutations to disrupt these two elements within the 23-nt ISS (Δ10F4) and assessed whether these mutations have any effect on exon 3 inclusion (Fig. 6A). Remarkably, an increase in exon 3 inclusion was observed in the Δ10F4 minigene with mutated poly-U tracts and the GGGG motif, when compared to Δ10F. Crucially, the extent of exon 3 inclusion was similar when compared to the Δ10F3 minigene, which has the 23-nt ISS removed (Fig. 6B). Collectively, these observations suggest that the poly-U tracts and the GGGG motif are essential elements within the 23-nt ISS that limit inclusion of exon 3.

### HnRNP H and hnRNP F do not regulate inclusion of *BIM* exon 3

We next investigated whether hnRNP H and hnRNP F were involved in repressing the inclusion of exon 3 via the GGGG motif. We used at least two different siRNA duplexes to downregulate each protein individually or both proteins simultaneously. Successful reduction of hnRNP H and F transcript levels was confirmed using real-time RT-PCR (Fig. 7A and 7B). Western blotting also verified the downregulation of hnRNP H protein levels upon siRNA treatment (Fig. 7C). Intriguingly, one of the siRNAs targeting hnRNP F (siF2) led to a 2-fold upregulation of hnRNP H transcripts, which suggest that knockdown of hnRNP F may cause a compensatory increase in hnRNP H levels (Fig. 7A). An increase in endogenous *BIM* exon 3 inclusion was not detected when either hnRNP H or F was depleted (Fig. 7D). Surprisingly, a modest decrease in exon 3 inclusion was observed when hnRNP H and F were depleted together when compared to control siRNA (Fig. 7D). Taken together, these observations strongly suggest that hnRNP H and F do not repress inclusion of *BIM* exon 3.

### Knockdown of hnRNP C enhances the inclusion of *BIM* exon 3 irrespective of the GGGG motif and the poly-U tracts within the 23-nt ISS

To determine whether hnRNP C is involved in repressing *BIM* exon 3 inclusion, we downregulated this protein using three different siRNA duplexes. Western blotting showed that all three siRNA duplexes substantially reduced the levels of the two major isoforms of hnRNP C (Fig. 8A). In addition, we observed a significant increase in inclusion of endogenous *BIM* exon 3 upon hnRNP C knockdown (Fig. 8B). These results show that hnRNP C functions as a repressor of *BIM* exon 3.

We next sought to elucidate whether hnRNP C represses *BIM* exon 3 via the poly-U tracts and the GGGG motif within the 23-nt ISS that we have identified earlier. To this end, we silenced hnRNP C using siRNAs and then nucleofected K562 cells with either Δ10F or Δ10F4 (Fig. 8C). Upon hnRNP C knockdown, inclusion of exon 3 was increased by at least 10-fold when K562 cells were nucleofected with the Δ10F minigene when compared to cells nucleofected with control siRNA (Fig. 8D). However, this effect was not significantly reduced when hnRNP C-depleted K562 cells were nucleofected with the Δ10F4 minigene. Collectively, we conclude that the repression of *BIM* exon 3 inclusion by hnRNP C is not mediated by the GGGG motif and the poly-U tracts within the 23-nt ISS.

### Downregulation of PTBP1, but not hnRNP C, inhibits imatinib-induced apoptosis

**Figure 9:**
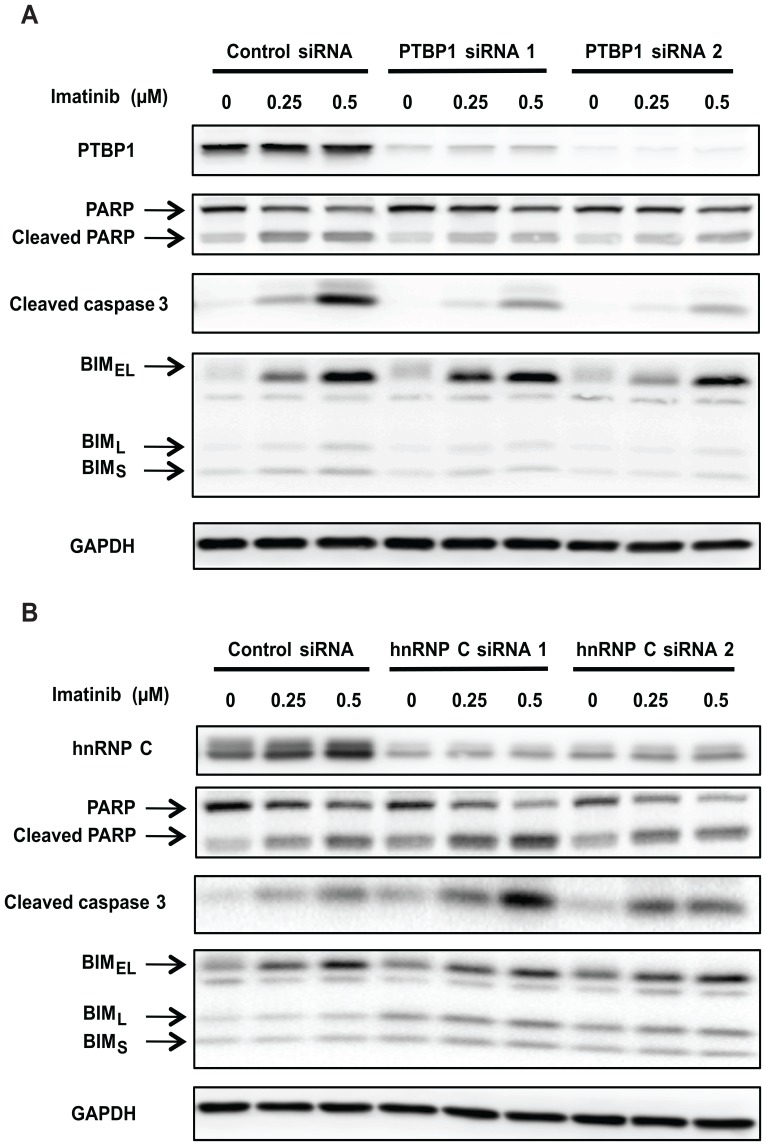
SiRNA-mediated knockdown of PTBP1, but not hnRNP C, inhibits imatinib-induced apoptosis in CML cells. (A) K562 cells were nucleofected with either control or PTBP1-specific siRNA duplexes. 24 hours later, these cells were treated with increasing doses of imatinib (0, 0.25, 0.5 µM). The cells were harvested after another 24 hours and western blot was performed to determine the protein levels of PTBP1, PARP, cleaved caspase 3 and BIM. Equal loading in each lane was determined by blotting of GAPDH. (B) The same experiment was performed as described in (A). The only difference was that siRNA duplexes against hnRNP C were used instead of PTBP1-specific siRNA duplexes.

Since downregulation of either PTBP1 or hnRNP C promoted the inclusion of *BIM* exon 3, which does not encode for the pro-apoptotic BH3 domain, we hypothesized that downregulation of either protein could suppress induction of BH3-containing BIM proteins, such as BIM_EL_, BIM_L_ and BIM_S_, and impair induction of apoptosis by TKIs such as imatinib. Consistent with our hypothesis, the downregulation of PTBP1 diminished imatinib-induced apoptosis in the K562 CML cell line, as indicated by the reduced expression of apoptotic markers like cleaved poly (ADP-ribose) polymerase (PARP) and cleaved caspase 3 (Fig. 9A). Furthermore, downregulation of PTBP1 also impaired the upregulation of BIM_L_ and BIM_S_ (Fig. 9A), which are more potent inducers of apoptosis than BIM_EL_ [25]. Interestingly, impaired induction of BIM_EL_ was not observed consistently when we used two different siRNA duplexes to knockdown PTBP1 (Fig. 9A).

The effects of downregulating hnRNP C on imatinib-induced apoptosis were also investigated. However, unlike PTBP1, siRNA-mediated knockdown of hnRNP C promoted the induction of imatinib-induced apoptosis as indicated by the increased expression of cleaved PARP and cleaved caspase 3 when compared to K562 cells that were nucleofected with control siRNA (Fig. 9B). Intriguingly, downregulation of hnRNP C slightly increased the induction of BIM_L_ and BIM_S_ upon treating K562 cells with increasing doses of imatinib (Fig. 9B).

## Discussion

Our earlier work demonstrated that a 2,903-bp deletion polymorphism within intron 2 of the *BIM* gene promotes the inclusion of exon 3 over exon 4, leading to a greater expression of BIM isoforms lacking the pro-apoptotic BH3 domain [16]. These results suggest that the 2,903-nt polymorphic fragment harbors *cis*-acting elements that regulate *BIM* pre-mRNA splicing by repressing inclusion of *BIM* exon 3. In this study, we describe some of the *cis*-acting elements within the 2,903-nt polymorphic fragment that repress inclusion of exon 3, as well as identify two splicing factors that regulate inclusion of *BIM* exon 3. To our knowledge, this study is the first to identify *cis*-acting elements that regulate *BIM* splicing. Our deletion analysis reveals that there are numerous redundant elements along the 2,903-nt polymorphic fragment that repress exon 3 inclusion. In addition, we could also define a minimal sequence that is sufficient but not necessary for repressing exon 3 inclusion to the last 322-nt comprising +2,582 to +2,903 of the polymorphic fragment (Fig. 1). From these results, we propose that the 2,903-nt polymorphic fragment contains multiple binding sites for splicing repressors, forming a silencing zone upstream of exon 3 that represses inclusion of the exon. The possibility of a long-range secondary structure within the 2,903-nt fragment that represses exon 3 is not supported by our findings, since large deletions on either side of the fragment do not significantly affect exon 3 inclusion (Fig. 2). Pending the identification of all individual ISSs within the 2,903-nt region, this fragment is the largest region with net splicing silencing activity described so far.

Regulation of exon inclusion can also be mediated by a mechanism which consists of splicing silencers juxtaposing or overlapping with splicing enhancers, or counteracting enhancers from a distance [36], [37]. This mechanism of splicing regulation has been described in fibroblast growth receptor 2 (*FGFR2*), human immunodeficiency virus and bovine papillomavirus transcripts, and human immunodeficiency virus tat exon 3, to name a few [36], [38]–[40]. In these systems, it has been proposed that the association of splicing repressors with the silencers represses exon inclusion by directly antagonizing the enhancers. However, we found no evidence that the 2,903-nt polymorphic fragment utilizes this mechanism to repress exon 3 inclusion, because our deletion analyses did not reveal any significant enhancer activity (Fig. 1 and 2).

There is growing evidence demonstrating that SNPs can modulate pre-mRNA splicing [41]–[43]. It has been estimated that allele-specific splicing affects the expression of around 20% of alternatively spliced genes [44]. The dbSNP at UCSC Genome Browser shows at least 13 SNPs within the 2,903-nt polymorphic fragment. Our finding of numerous redundant *cis*-acting elements along the 2,903-nt fragment that repress *BIM* exon 3 suggests that each individual SNP may not play a significant role in the regulation of exon 3 inclusion.

Detailed analysis of the last 322-nt of the polymorphic fragment revealed two regions (+2,582 to +2,662 and +2,823 to +2,903) that account for most of its silencing activity (Fig. 2). It has been shown that RNA secondary structure may cause *cis*-acting elements to become inaccessible to splicing factors thereby promoting exon skipping [45]. Since +2,582 to +2,662 and +2,823 to +2,903 are close to each other and to the 3′ splice site of exon 3, it could be possible that these two segments form a secondary structure that impairs inclusion of this exon. If this were true, removing +2,582 to +2,662 *or* +2,823 to +2,903 of the polymorphic fragment from the Δ10 minigene should abolish the structure and hence, lead to a greater inclusion of exon 3. However, partial and complete removal of +2,582 to +2,662 or +2,823 to +2,903 (Fig. 2, MUT1 and MUT5, Δ10A and Δ10B) failed to increase exon 3 inclusion as compared to Δ10C. These observations argue against the notion that +2,582 to +2,662 and +2,823 to +2,903 of the polymorphic fragment form a secondary structure that represses inclusion of exon 3, and is consistent with the presence of ISSs that act separately via binding of splicing repressors.

Computational analyses predicted three putative binding sites for PTBP1 within +2,582 to +2,662 of the polymorphic fragment, suggesting that PTBP1 could repress inclusion of *BIM* exon 3 by binding to these sites. Indeed, downregulation of PTBP1 using siRNAs led to a significant increase in the ratio of endogenous *BIM* transcripts containing exon 3 over exon 4 (Fig. 3), indicating that PTBP1 indeed functions as a repressor of exon 3. Here, removing the three putative PTBP1 binding sites from +2,582 to +2,662 did not significantly increase splicing of exon 3 (Fig. 4B). Furthermore, silencing of PTBP1 had no effect on splicing of *BIM* minigenes with or without the polymorphic fragment (Fig. 4C). Collectively, our results suggest that PTBP1 does not act via the polymorphic fragment to repress exon 3 inclusion. Since our minigenes do not contain all the sequences in introns 2 and 3, it is possible that PTBP1 may bind other *cis*-acting elements within *BIM* to repress exon 3 inclusion. Of note, PTBP1 has also been implicated in the regulation of cap-independent translation mediated by the internal ribosomal entry site [46], [47], as well as in the splicing regulation of transcripts encoding other splicing factors [48]. Therefore, it is also possible that PTBP1 represses exon 3 inclusion indirectly by changing the expression of other splicing factors.

In this study, we have identified a 23-nt ISS within +2,823 to +2,903 of the polymorphic fragment (Fig. 5). Mutational analysis revealed that the two poly-U tracts and a GGGG motif are critical elements within the ISS that repress the inclusion of *BIM* exon 3 (Fig. 6). HnRNP H and hnRNP F are two similar proteins that bind G-rich sequence motifs. Depending on the location of the G-rich motifs, hnRNP H either promotes or represses exon inclusion. If the G-rich motifs are located within exons or within intronic elements upstream of the 3′ splice site, then hnRNP H represses exon inclusion [49]–[52]. In contrast, hnRNP H enhances exon inclusion if it binds to G-rich motifs downstream of the 5′ splice site [53]–[56]. As the GGGG motif within the 23-nt ISS is relatively close to the 3′ splice site of *BIM* exon 3 (120 nts), we asked whether hnRNP H and hnRNP F played a role in repressing exon 3 inclusion. However, downregulation of hnRNP H and hnRNP F failed to significantly increase endogenous *BIM* exon 3 inclusion (Fig. 7D), suggesting that other splicing regulators bind to the GGGG motif within the 23-nt ISS. We also addressed whether hnRNP C regulates *BIM* exon 3 inclusion via the poly-U tracts within the 23-nt region [34]. We found that hnRNP C functions as a repressor of endogenous *BIM* exon 3 inclusion (Fig. 8B), but this repression was not dependent on the poly-U tracts (Fig. 8D). Thus, similarly to PTBP1, hnRNP C represses *BIM* exon 3 inclusion directly by binding to other sequences of the *BIM* transcript, or indirectly by regulating other factors.

The identification of PTBP1 and hnRNP C as repressors of *BIM* exon 3, although not acting via the polymorphic fragment, has potential clinical relevance. Our findings indicate that decreased expression of PTBP1 and hnRNP C could promote the expression of exon 3-containing BIM isoforms that lack the pro-apoptotic BH3 domain, thereby impairing the induction of BH3-containing BIM isoforms. Therefore, reduced expression of PTBP1 and hnRNP C could contribute to inferior responses to anticancer agents that rely on BIM expression, such as TKIs [57]. Indeed, when we used siRNAs to downregulate PTBP1, we observed diminished induction of imatinib-induced apoptosis in CML cells, as well as impaired induction of BIM isoforms that harbor the BH3 domain encoded in exon 4 (BIM_L_ and BIM_S_) (Fig. 9A). Interestingly, impaired induction of BIM_EL_ was not observed consistently when we knockdown PTBP1. One possible explanation could be that, unlike BIM_L_ and BIM_S_, BIM_EL_ is rapidly degraded by the proteasome pathway as a result of phosphorylation by extracellular signal-regulated kinases 1/2 (ERK 1/2) [58]–[60]. Therefore, even when the induction of exon 4-containing *BIM* transcripts is suppressed by the downregulation of PTBP1, the inhibition of ERK1/2 signaling by imatinib can still increase BIM_EL_ protein levels by suppressing BIM_EL_ turnover. Interestingly, a recent report showed that downregulation of PTBP1 sensitizes ovarian cancer cells to chemotherapeutic agents such as carboplatin or paclitaxel [61]. These findings appear contradictory to what we observed when we downregulated PTBP1 in CML cells. These conflicting observations could be accounted for by tissue-specific expression of their target transcripts or other splicing factors [62]. Therefore, downregulation of PTBP1 in ovarian cancer cells may not lead to changes in the splicing of *BIM* exons 3 and 4. Unexpectedly, downregulation of hnRNP C enhanced imatinib-induced apoptosis and promoted the induction of BIM_L_ and BIM_S_ (Fig. 9B). Because hnRNP C regulates the splicing of numerous other pre-mRNAs [34], we speculate that hnRNP C downregulation could promote the expression of splice variants in other transcripts that sensitize CML cells to imatinib-induced apoptosis.

Our deletion analyses of the *BIM* polymorphic fragment presented here depict a “coarse map” of the splicing silencers within this region, as a first step towards a deeper understanding of *BIM* exon 3 splicing. The identification of these regulatory elements is crucial for the development of ASOs that bind to and inhibit *cis*-acting elements to alter *BIM* splicing for therapeutic purposes. Increased BIM expression has been shown to contribute to increased cardiomyocyte and neuronal cell death following ischemia [11], [12]. Therefore, a potential therapeutic approach to reduce cell death is to decrease the expression of exon 4-containing *BIM* transcripts. This can be done by employing ASOs that bind to splicing silencers of exon 3. However, our findings suggest that ASOs targeting ISSs within the polymorphic fragment would have little or no effects on *BIM* splicing because of the silencer redundancy within this region. Instead, it may be more appropriate to design ASOs that bind to splicing enhancers of exon 4 to repress its inclusion. ASOs can also be used to promote the inclusion of *BIM* exon 4 to enhance the cell-killing effects of anticancer agents. However, the 2,903-nt fragment is not an ASO drug target for cancers because our deletion analyses did not reveal any significant enhancer activity. Instead, exon 4 inclusion could be increased by ASOs targeting enhancer sequences within or flanking exon 3. Increasing PTBP1 expression is another possible approach to enhance exon 4 inclusion. In any event, it is important to note that modulating the expression of a splicing factor will affect the splicing of numerous transcripts in both tumor and non-diseased cells. Therefore, the global effects of PTBP1 upregulation should be carefully assessed before PTBP1 could be considered a drug target to induce tumor-cell death. Finally, future studies should reveal the fine location of the ISSs within the polymorphic fragment, as well as more splicing factors that regulate *BIM* exon 3 splicing.
